# Supplementary figures and images for: CD177 is a novel IgG Fc receptor and CD177 genetic variants affect IgG-mediated function
Source: Front Immunol. 2024 Jul 26;15:1418539. doi: 10.3389/fimmu.2024.1418539 (PMC11316256; doi:10.3389/fimmu.2024.1418539)

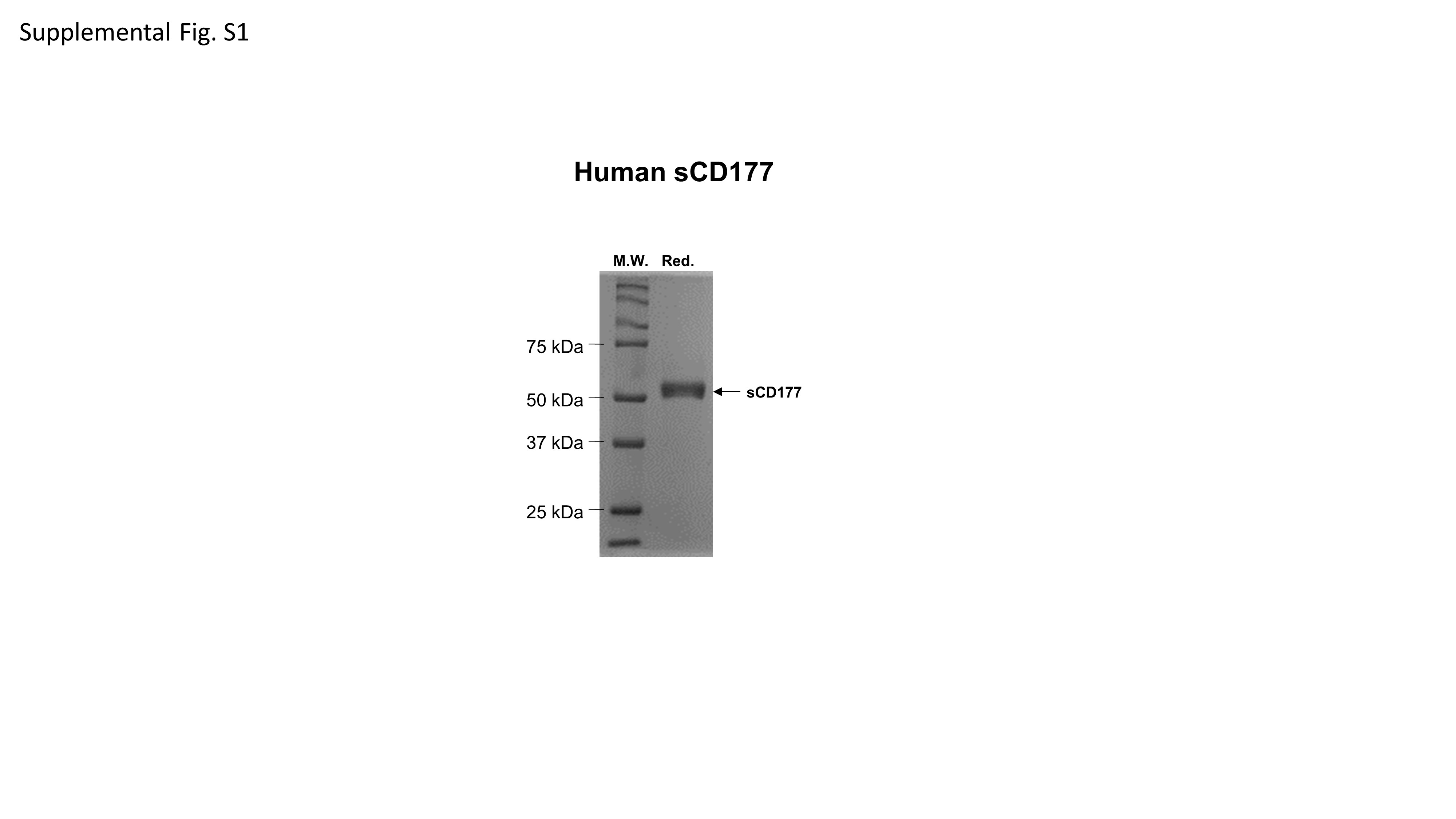

Supplement: Supplementary file 1 [file Image_1.jpeg]

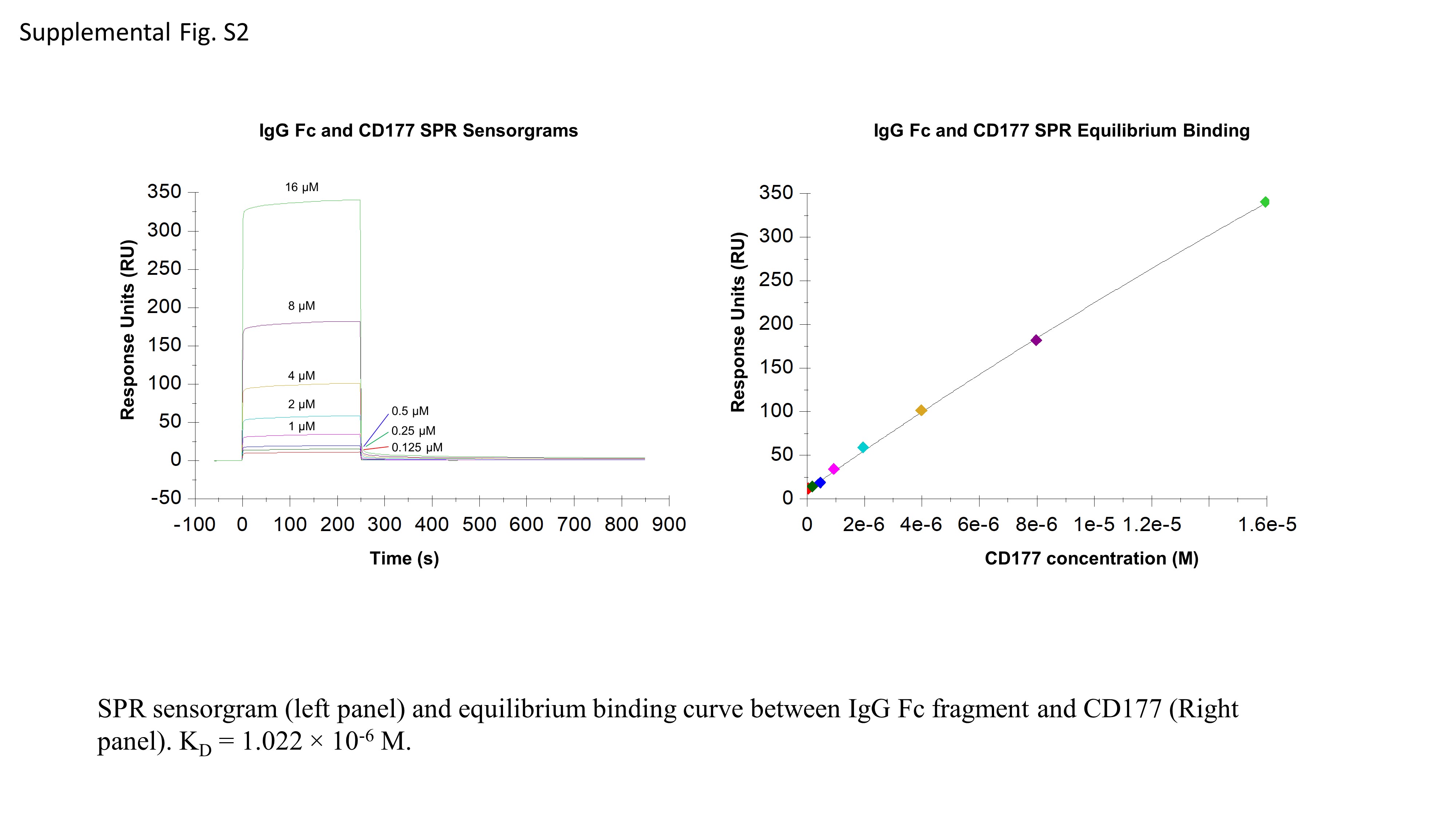

Supplement: Supplementary file 2 [file Image_2.jpeg]

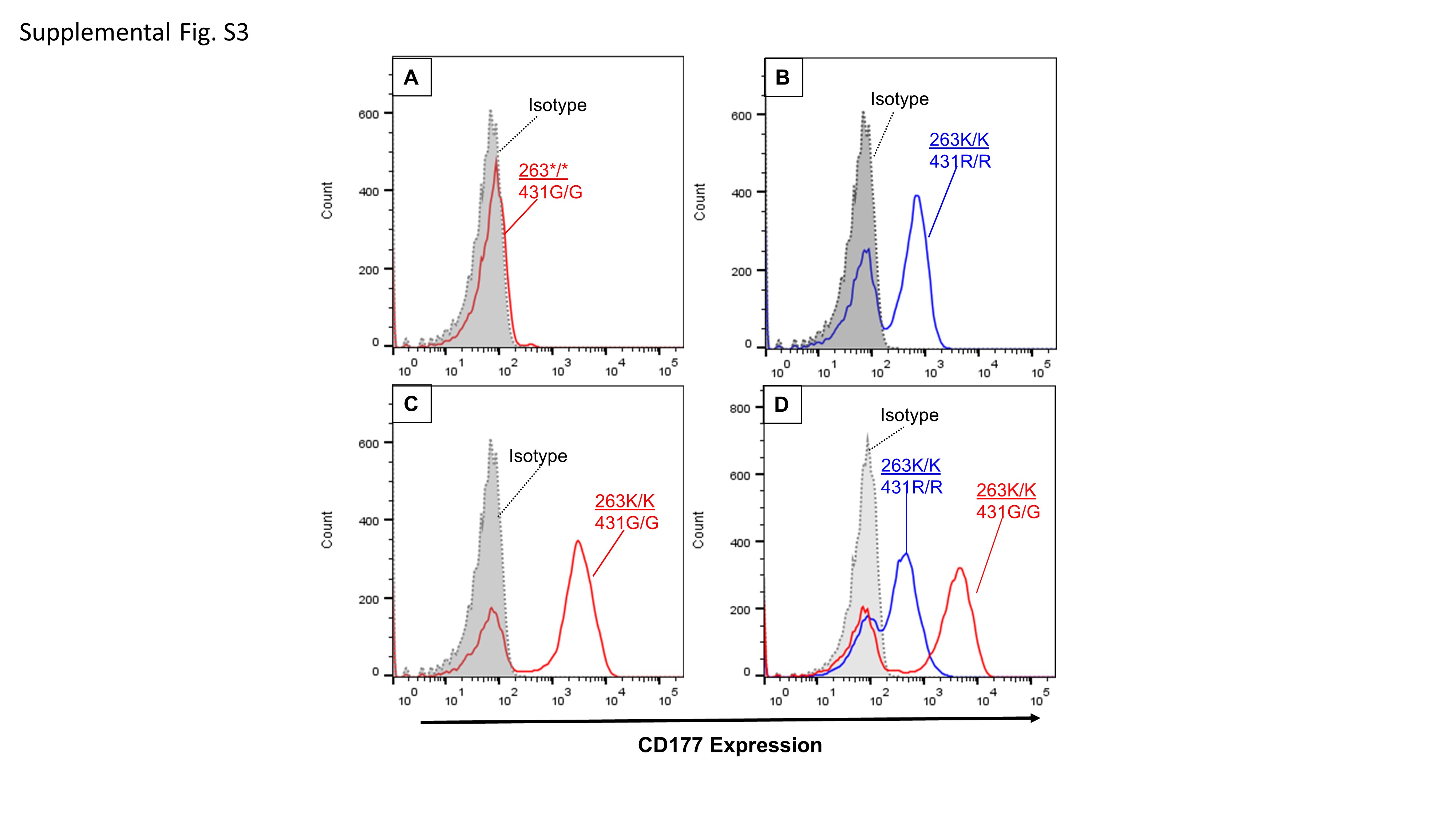

Supplement: Supplementary file 3 [file Image_3.jpeg]
